# ASF1B promotes cervical cancer progression through stabilization of CDK9

**DOI:** 10.1038/s41419-020-02872-5

**Published:** 2020-08-26

**Authors:** Xinjian Liu, Jingwei Song, Yenan Zhang, Huiquan Wang, Hongzhi Sun, Xiaomin Feng, Min Hou, Guo Chen, Qi Tang, Minjun Ji

**Affiliations:** 1 Department of Pathogen Biology, Nanjing Medical University, 211166 Nanjing, China; 2 Key Laboratory of Antibody Technique of National Health Commission of China, Nanjing Medical University, 211166 Nanjing, China; 3 Laboratory Medicine Center, The Second Affiliated Hospital of Nanjing Medical University, 210000 Nanjing, China; 4 The Affiliated Obstetrics and Gynecology Hospital of Nanjing Medical University, 210004 Nanjing, China; 5 Department of Medical Biochemistry and Molecular Biology, School of Medicine, Jinan University, 510632 Guangzhou, China

**Keywords:** Tumour biomarkers, Genetics research

## Abstract

Cervical cancer (CC) is one of the most deadly cancers in women, its current treatments still result in poor outcomes and developing the novel targets and therapeutic strategies are urgently needed. Recent studies have shown that anti-silencing function 1B (ASF1B) might be used as a new proliferation marker for cancer diagnosis and prognosis. However, the expression and function of ASF1B in cervical cancer remain unclear. Here, we induced ASF1B knockdown and overexpression in cervical cancer cell lines and detected the biological behavior changes in vitro. Furthermore, we established two murine models using stable ASF1B-shRNA HeLa cells or normal HeLa cells following AAV-shRNA-ASF1B administration to evaluate how suppression of ASF1B affects tumor growth. We showed that ASF1B functions as an oncogene in cervical cancer cells. Silence of ASF1B suppressed cervical cancer cell growth in vitro and in vivo, while, ASF1B overexpression accelerated cancer cell proliferation. Furthermore, ASF1B deficiency induced cell cycle arrest and apoptosis. Mechanistically, we found that ASF1B formed stable complexes with cyclin-dependent kinase 9 (CDK9), and positively regulated CDK9 stabilization. Taken together, tumorigenic ASF1B could be targeted to suppress cervical cancer tumor growth by inducing apoptotic cell death.

## Introduction

Cervical cancer is the fourth leading cause of cancer death in the world and the second most common female malignant tumor, with a mortality rate next only to only that of breast cancer^1–3^; there are ~470,000 new cases of CC each year, and the disease poses a great threat to the life of ~276,000 females^4^. Among these cases, approximately 90% occur in developing countries, and more than 50,000 females succumb to cervical cancer each year in China^5,6^. Our current understanding of cervical cancer is that persistent human papillomavirus (HPV) infection contributes to tumor establishment^7–10^. However, HPV infection alone is not sufficient for tumor development, and host genetic factors may also be involved in cervical cancer pathogenesis^11^. Although clinical cervical cancer treatments, including surgery, chemotherapy, and radiotherapy, are usually performed, poor effects of clinical chemotherapeutic treatments have been observed^12^. Thus, the tumorigenesis mechanism of cervical cancer should be further studied, and new drug targets and treatment strategies should be sought.

As mentioned above, the molecular basis of cancer involves host genetic factors mutations^13,14^. Abnormal gene expression, including gene silencing or overexpression, is associated with variations in DNA methylation and aberrant histone post-translational modifications^15–17^. The deregulation of chromatin regulators has been shown to be involved in the occurrence and progression of cancer, including histone variant proteins, histone chaperone proteins, histone modifying enzymes, effector proteins and chromatin remodeling proteins^18–20^. Histone H3–H4 chaperone anti-silencing function 1 (ASF1) is an important histone chaperone protein that plays a role in the chromatin-based progression of cellular DNA replication, DNA damage repair, and transcription regulation^21,22^. ASF1 exists as two paralogs: ASF1A and ASF1B. ASF1A contributes primarily to DNA repair and cell senescence, while ASF1B is preferentially involved in cell proliferation^22,23^. Interestingly, Armelle et al. revealed that a high mRNA level of ASF1B was correlated with clinical data and disease outcome in breast cancer, and the researchers proposed that ASF1B might be used as a new proliferation marker for breast cancer diagnosis and prognosis^24^.

Here, we induced ASF1B knockdown and overexpression in cervical cancer cell lines and detected the biological behavior changes in vitro. Furthermore, we established two murine models using stable ASF1B-shRNA HeLa cells or normal HeLa cells following AAV-shRNA-ASF1B administration to evaluate how suppression of ASF1B affects tumor growth. Our data implied that ASF1B was closely associated with proliferation, migration, and anti-apoptosis in cervical cancer cells and might be considered a novel therapeutic target and prognostic indicator in cervical cancer patients.

## Materials and methods

### Patients and tissue samples

We collected cervical cancer tumors and corresponding adjacent tissue samples from 50 patients who underwent tumor resection (The Affiliated Obstetrics and Gynecology Hospital of Nanjing Medical University). The protocol was approved by Nanjing Medical University Ethics Committee (No. 2019923). All tumors and matched non-tumor specimens were diagnosed by pathology. No patients received chemotherapy and/or radiotherapy before surgery. The resected specimens were subjected to qPCR (12 samples) and to immunohistochemistry (IHC, 50 samples) using an anti-human ASF1B antibody (Invitrogen, US).

### Cell lines, cell culture, plasmid, reagents

Human CaSki, HeLa, and HaCaT cells were obtained from Procell (Wuhan, China). The cell lines were maintained in Dulbecco’s modified Eagle’s medium (DMEM, Life) supplemented with 100 U/ml penicillin, 100 μg/ml streptomycin, 2 mmol/l l-glutamine, 10 mmol/l HEPES, and 10% fetal bovine serum (FBS) at 37 °C in a 5% CO_2_ humidified atmosphere. The cell lines have been confirmed as mycoplasma contamination free by Procell (Wuhan, China).

### Total RNA extraction and qPCR

Total RNA was extracted using TRIzol reagent (Thermo Fisher, Shanghai, China) and reverse transcribed into cDNA using PrimeScript™ RT Master Mix (Takara, Dalian, China). qPCR was performed with a Roche Detection System (Roche) in a 20-µl reaction mixture containing SYBR Green I. The expression data were normalized to the housekeeping gene GAPDH rRNA and were analyzed using the 2^-ΔΔCT^ method.

### Knockdown and overexpression of ASF1B in stable cell lines

ASF1B-shRNAs were designed, synthesized, and annealed to form double-strand DNA fragments that were cloned into the pGPU6/GFP/Neo vector (Gene Pharma, Shanghai, CN). The recombinant plasmid was transfected into human CaSki and HeLa cells with Lipofectamine®2000 (Invitrogen, US). The cells were incubated at 37 °C in complete Opti-MEM medium containing 1200 μg/ml (HeLa) or 500 μg/ml (CaSki) G418 (Hanbio Biotechnology) for 30 days to select positive cells. A limited dilution strategy was performed to acquire the single cell clones. Total RNA from each clone was extracted, and qPCR was performed to explore whether the ASF1B gene was silenced. Protein expression levels were detected by western blot. The positive cells were selected and named ASF1B-shRNA cells.

The ASF1B overexpression plasmid pcDNA3.1-ASF1B was transfected into human CaSki and HeLa cells using Lipofectamine®2000 (Invitrogen). The overexpression level of ASF1B was identified by qPCR and western blot. Positive cells were selected and expanded to perform the following experiments.

### AAV-shRNA-ASF1B construction and AAV virus packaging

The AAV-shRNA-ASF1B plasmid was constructed by Hanbio Biotechnology. Packaging of AAV-shRNA-ASF1B was performed as described before^25^. In brief, HEK 293 cells were transfected with a plasmid mix of pAAV-shRNA-ASF1B, GV388, pH21, and pHELPER (molar ratio: 0.5: 0.5: 1: 1) using Lipofectamine®2000. Twenty-four and forty-eight hours after transfection, the culture supernatants were collected and purified using Lenti-X™ Concentrator (Clontech, US). Viral titers were then determined using qPCR.

### Animal experiments

Female nude mice (6 weeks old) were purchased from Beijing Vital River Laboratory Animal Technology Co., Ltd. All experiments with mice were performed according to a protocol approved by the Nanjing Medical University Animal Care and Use Committee (Approval number: IACUC-1705003). All mice received humane care according to the criteria outlined in the “Guide for the Care and Use of Laboratory Animals”.

Model 1: Mice were divided randomly into two groups (*n* = 5/group): the ASF1B-shRNA group and the scrambled shRNA control group. Next, 5 × 10^6^ stable ASF1B-shRNA HeLa cells or corresponding scrambled cells with 15% Matrigel (BD, #354248) in 100 µl PBS were injected subcutaneously into the flank of each nude mouse. Tumor sizes were measured by calipers every 3 days. The tumor volumes were calculated as follows: tumor volume (mm^3^) = 1/2 × length × width^2^.

Model 2: Subcutaneous tumors following AAV-shRNA-ASF1B administration were also investigated. In brief, nude mice were also divided randomly into two groups (*n* = 5/group): the AAV-shRNA-ASF1B group and the scrambled AAV control group. Next, 5 × 10^6^ HeLa cells with 15% Matrigel in 100 µl PBS were injected subcutaneously into the flank of each nude mouse. AAV-shRNA-ASF1B or scrambled AAV-shRNA at a dose of 5 × 10^11^ viral particles was injected directly into the tumors on days 12, 15, 18, and 27. Tumor sizes were measured every 3 days. Seven weeks later, all mice were sacrificed, and the tumors were excised, harvested, weighed, and fixed in formalin for at least 24 h^26–28^.

### Western blot analysis

To lyse cells, cell pellets were resuspended in cold lysis protein extraction reagent (Thermo Fisher Scientific, Inc) with a protease inhibitor cocktail (Sigma); the protein was quantified and then used to perform western blot as previously described^29^. Antibodies against ASF1B, cyclin A1, cyclin B1, cyclin D1, cleaved caspase 3, Bcl-2, Bax, and β-actin were purchased from Cell Signaling (Danvers, MA). Mouse anti-CDK9 antibody was obtained from Novus (Centennial, CO) and rabbit anti-CDK9 were purchased from Cell signaling (Danvers, MA).

### Cell viability assay

Cell viability analysis was performed using a Cell Counting Kit-8 (CCK-8; Beyotime Institute of Biotechnology, Shanghai, China). Briefly, 2 × 10^3^ cells were seeded into 96-well plates. At 24 h, 48 h and 72 h, 10 µl CCK-8 solution was added to each well, and the cells were incubated for 3 h at 37 °C. Then, the absorbance was measured using a microplate reader (Bio-tech, CA, USA) with a 450 nm wavelength.

### Colony formation assay

Cells grown to 90% confluence were harvested and seeded in 10-cm plates at 300 cells per plate. Fourteen days later, after washing with PBS, the cells were stained with a 0.05% crystal violet solution for imaging and colony counting.

### Wound healing assay

Cells were seeded in 6-well plates until their growth confluence reached 90%. Next, 2-mm scratches were performed using a 200-µl pipette tip. The cell-free gaps were measured over time with an optical microscope (Zeiss).

### Invasion assay

A total of 50 µg/cm^2^ reconstituted Matrigel (Sigma-Aldrich) was used to coat polycarbonate filters (8 µm; Corning, NY, USA). Next, 2 × 10^3^ cells were seeded into the upper chamber with 200 µl serum-free DMEM. DMEM with 10% FBS was added to the lower chamber, and the cells were cultured at 37 °C in a 5% CO_2_ humidified atmosphere. Twenty-four and forty-eight hours later, non-invading cells were removed, and the cells on the lower surface were counted microscopically^30^.

### Flow cytometry for analysis of the cell cycle and apoptosis

To perform cell cycle assays, cells were fixed with 70% EtOH for 15 min and then washed with PBS. Cell pellets were suspended in 500 µl PI solution (BD Biosciences, San Jose, CA) and incubated for 40 min at 37 °C. Before washing, 3 ml PBS was added. The cells were pelleted, and the supernatant was removed; then, the pellet was suspended in 500 µl PBS for flow cytometry analysis.

Cell apoptosis analysis was performed with a PE Annexin V Apoptosis Detection Kit (Beyotime, Shanghai, China) according to the manufacturer’s protocol. In brief, after washing with cold PBS twice, the cells were resuspended in binding buffer. A total of 1 × 10^5^ cells (100 µl) were transferred to the flow tube, and 5 µl of PE Annexin V and 5 µl 7-AAD were added. After gentle vortexing and incubation for 15 min at room temperature in the dark, another 400 µl binding buffer was added and then analyzed by flow cytometry.

### Immunohistochemistry

Tumor tissue sections were harvested and treated as previously described^31^. Primary antibodies for anti-human ASF1B (1:100), anti-mouse ASF1B, Ki67, and CD31 (1:100) were purchased from Cell Signaling Technologies. An ImmPRESS™ HRP Anti-Rabbit IgG (Peroxidase) Polymer Detection Kit, raised in horse (Vector Laboratories), was used for the secondary antibody. ImmPACT DAB Peroxidase (HRP) Substrate was used for detection, and hematoxylin (Vector Laboratories) was used as a counterstain.

### Protein–protein interaction studies

Co-IP was conducted as previously described^32^. Briefly, whole-cell extracts of Flag-ASF1B-transfected HeLa cells were prepared, and immunoprecipitation was performed by adding 20 µl of anti-Flag affinity sepharose or normal iso-type antibody and incubating overnight at 4 °C. Finally, the immune complex was detected by Western blot. As controls, samples without treatment were used. Then, the Co-IP product was separated by SDS-PAGE. The whole gel was subjected to silver staining and then liquid chromatography-mass spectrometry (LC-MS) analysis^33^.

The LC-MS analysis results indicated that cyclin-dependent kinase 9 (CDK9) might be involved in the progression of cervical tumors. To further confirm this hypothesis, Co-IP was performed using an anti-ASF1B antibody or anti-CDK9 antibody. Antibodies against ASF1B and CDK9 for Co-IP were purchased from Novus Biologicals (Colorado, US). To test the protein stability, cells were incubated with 20 μg/ml Cycloheximide (CHX, Sigma-Aldrich) for the indicated periods of time. To further determine whether ASF1B promote the proteasomal stabilization of CDK9, ASF1B-shRNA HeLa cells or control cells were treated with or without proteasome inhibitor MG132. The treated cell proteins were extracted for western blot.

### Cell immunofluorescence staining

For double immunofluorescence staining, cells grown on coverslips were prepared and fixed in 4% paraformaldehyde for 15 min. Then, the cells were treated with 0.3% Triton X-100 for 20 min and blocked with 10% normal goat serum for an hour. The cells were then incubated with primary antibodies (1:100), including rabbit anti-human ASF1B mAb (Cell Signaling, Danvers, MA) and mouse anti-human CDK9 mAb (Novus Biologicals, Colorado, USA) at 4 °C overnight. The cells were washed with PBS and then incubated with the appropriate Alexa-488- or TRITC-conjugated secondary antibodies (1:200; Proteintech, Chicago, USA) at room temperature for 60 min. The cells were counterstained with DAPI and photographed using a Zeiss Axio Imager and microscope LSM710 (Carl Zeiss, Jena, Germany).

### Statistics analysis

Paired data were analyzed using a 2-tailed paired Student’s *t* test. A *p* value of < 0.05 was considered significant.

## Results

### Patients’ characteristics

Primary characteristics of cervical cancer patients were shown in Table 1. The median age of patients was 53.5 years, the age range was from 26 to 73 years old and 82% (41/50) were over 35 years. Histopathological results revealed that 98% (49/50) of cases were of cervical squamous cell carcinoma, and 2% (1/50) were of adenocarcinoma. In line with the FIGO staging, the clinical staging was carried out: 27 cases were stage I and 18 cases were stage II. According to the WHO classification, the pathological grades were classified into groups with 2 cases (4%) highly differentiated carcinoma, 39 cases (78%) moderately differentiated, and 9 cases (18%) poorly differentiated.

**Table 1:** Association between ASF1B expression and clinicopathologic parameters of cervical cancer patients.

		ASF1B expression					
Characteristic	Total	low		high		Statistics	*P*
**Age, years**	50		Mean		Mean		
≤35	5	3	48.24	2	52.12	1.204	0.2344
>35	45	30		15			
**FIGO stage**	45						
I	27	21		6		0.6818	0.4090
II	18	12		6			
**Tumor size (cm)**	50						
<4	44	30	1.700	14	3.029	2.652	0.0108*
≥4	6	3		3			
**Deep stromal invasion**Δ	34						
<2/3	9	7		2			0.6921
≥2/3	25	17		8			
**Lymphovascular space invasion**Δ	25						
Negative	18	10		8			0.0573
Positive	7	7		0			
**Nerve invasion**	49						
Negative	41	27		14		0.2546	0.6138
Positive	8	6		2			

*NOTE:* These four clinico pathologic parameters, including FIGO stage, Deep stromal invasion, Lymphovascular space invasion and nerve invasion, have some missing samples. Δ: Fisher’s exact test (*n* < 40)

### ASF1B expression was upregulated in cervical cancer tissues and stable cell lines

**Fig. 1:**
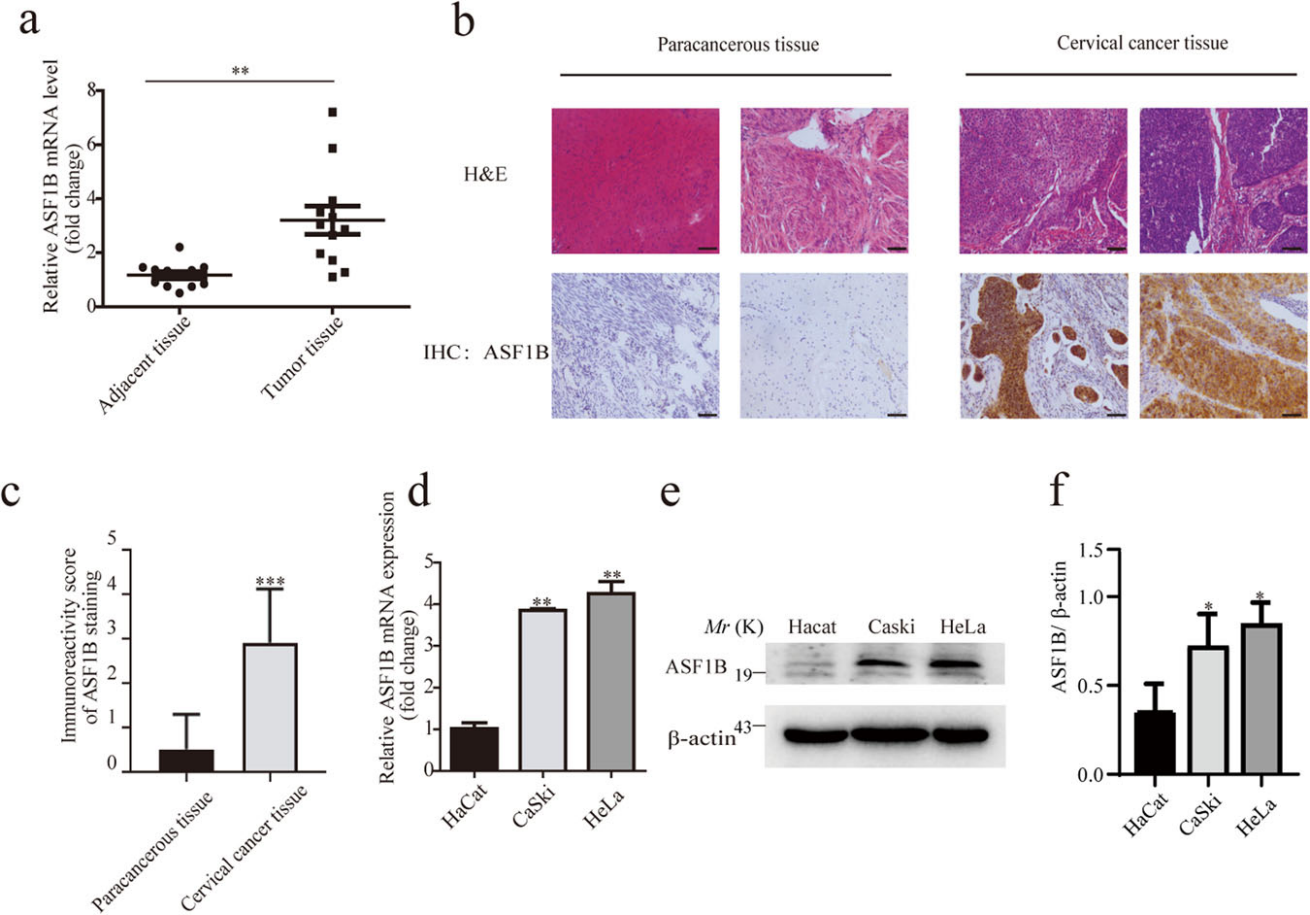
ASF1B expression in cervical tumors, adjacent tissues and human cervical cancer cell lines. **a** The expression level of ASF1B was assessed in paired cervical cancer and adjacent tissues from 12 patients by qPCR. **b** Pairs of cervical cancer tissue and paracancerous tissue samples from 50 patients were tested by H&E and immunohistochemistry. **c** Immunoreactivity score of ASF1B staining from the immunohistochemistry results. **d** Levels of ASF1B mRNA expression in cervical cancer cell lines (CaSki and HeLa) and a control cell line (HaCat). **e** The protein expression of ASF1B in CaSki, HeLa and HaCat cells by western blot. A higher level of ASF1B was detected in CaSki and HeLa cells than that in HaCat cells. **f** Quantification of the results in (**e**). Data are mean ± SEM, *n* = 3, and two-tailed unpaired Student’s *t* test was used. ***p < 0.001.

ASF1B has been shown to promote breast cancer, prostate cancer, cell renal cell carcinoma^24,34,35^. However, its expression level and role in cervical cancer remain to be elucidated. To investigate the role of ASF1B in cervical cancer tumor progression, the expression level of ASF1B was assessed with paired cervical tumors and adjacent tissues from 12 patients by qPCR. Compared with that in adjacent tissues, ASF1B was remarkably increased in tumor tissues (*p* < 0.05, *n* = 12) (Fig. 1a). To further evaluate the level of ASF1B, cervical cancer tissue and corresponding adjacent tissue samples from 50 patients were tested by immunohistochemistry. As shown in Fig. 1b, c, positive staining for ASF1B was stronger in cervical cancer tissues than in adjacent tissues (*p* < 0.05). We also explored the correlation between ASF1B level and clinical cervical tumor patients’ features^36^. Upregulation of ASF1B was positively correlated with tumor size (*p* = 0.0108, Table 1). These data indicated the possible involvement of ASF1B cervical cancer cells proliferation.

Next, we examined whether ASF1B was overexpressed in different cervical cancer cell lines: CaSki and HeLa cells. As shown in Fig. 1d, the qPCR analysis results showed that ASF1B mRNA levels were higher in CaSki cells and HeLa cells than in HaCaT cells, which is a spontaneously transformed aneuploid immortal keratinocyte cell line from adult human skin^37^. Western blot results further indicated that the level of ASF1B protein expression was much higher in CaSki cells and HeLa cells than in HaCaT cells (Fig. 1e, f). All these data illustrated the differential expression of ASF1B in distinct cervical cancer cells.

### Knockdown or overexpression of ASF1B was mediated by shRNA or recombinant plasmid in cervical cancer cell lines

**Fig. 2:**
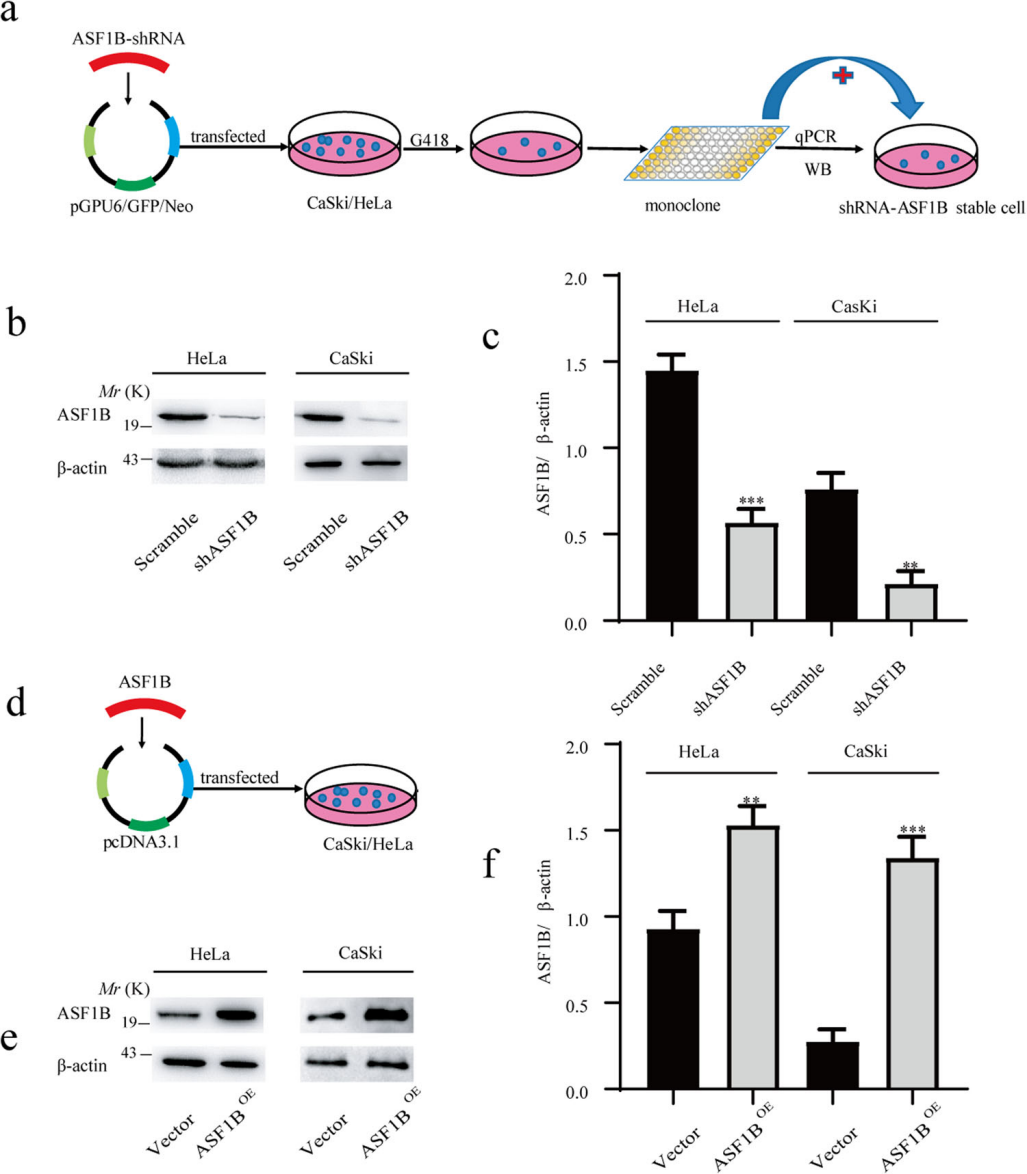
Knockdown or overexpression of ASF1B in cervical cancer cells. **a** A total of 1 × 10^5^ CaSki or HeLa cells were seeded in each well of a 6-well plate, and the recombinant plasmid ASF1B-shRNA was transfected into cells with Lipofectamine®2000. The cells were incubated in complete Opti-MEM medium containing G418 for 30 days to select positive cells. A limited dilution strategy was performed, and the positive cell clones were selected. **b** ASF1B protein expression in stable ASF1B-shRNA-cells. Western blot detecting lower ASF1B protein levels in stable ASF1B-shRNA-CaSki or ASF1B-shRNA-HeLa cells than those in control cells. **c** Quantification of the results in (**b**). Data are mean ± SEM, *n* = 3, and two-tailed unpaired Student’s *t* test was used. ***p < 0.001. **d**. Establishment of ASF1B overexpression in CaSki or HeLa cells with overexpression technology. pcDNA3.1-ASF1B was transfected into human CaSki and HeLa cells using Lipofectamine®2000. **e** Western blot results showed that observable ASF1B protein was detected in ASF1B-CaSki and HeLa cells relative to that in control cells. **f** Quantification of the results in (**e**). Data are mean ± SEM, *n* = 3, and two-tailed unpaired Student’s *t* test was used. ***p < 0.001.

To study the role of ASF1B in cervical cancer, shRNA technology was used to knock down ASF1B. Oligonucleotides were synthesized and then annealed to make a double-stranded DNA fragment that was inserted into the pGPU6/GFP/Neo vector. This recombinant vector or scrambled vector was transfected into CaSki cells and HeLa cells with Lipofectamine®2000 (Invitrogen). With limited dilution strategies, a single cell clone was selected (Fig. 2a), and the mRNA and protein levels were detected by qPCR and western blot. The results from western blot (Fig. 2b, c) validated that the ASF1B level was reduced and that ASF1B knockdown was successful in CaSki cells and HeLa cells; thus, the corresponding stable ASF1B knockdown cell lines were established.

Ectopic expression was also induced by plasmid-mediated technology. pcDNA3.1-ASF1B was transfected into human CaSki and HeLa cells (Fig. 2d). Increased ASF1B mRNA and protein expression levels were detected by western blot (Fig. 2e, f).

### Knockdown of ASF1B inhibited cervical cancer cell proliferation

A CCK-8 assay was conducted to examine cancer cell proliferation. The results showed that ASF1B knockdown led to a significant reduction in cell viability (Fig. S1a, S1b). A colony formation assay was also used to investigate the effect of ASF1B on cervical cancer cell growth. The images showing colony formation and clone number demonstrated that ASF1B knockdown significantly suppressed HeLa cells and CaSki cells (*p* < 0.01, Fig. S1c, S1d). In contrast, ectopic expression of ASF1B significantly accelerated cancer cell proliferation as evidenced by CCK-8 assay (Fig. S1e, S1f) and induced cell growth according to colony formation assay (Fig. S1g, S1h). These results suggested that ASF1B alteration significantly inhibits cervical cancer cell proliferation and anchorage-free colony formation.

### Knockdown of ASF1B suppressed cervical cancer cell migration

Wound healing assays were performed to test whether the ASF1B gene impacts cervical cancer cell motility. Representative images and the quantified results indicated that ASF1B knockdown significantly suppressed cancer cell motility (Fig. S2a, S2b). To further examine the effect of ASF1B on cell invasion, a transwell assay was also performed. Compared with the scrambled cervical cancer cells, ASF1B-shRNA-transfected cervical cancer cells showed significantly decreased invasiveness (*p* < 0.01, Fig. S2c, S2d). Wound healing and transwell assay results showed that knockdown of ASF1B significantly inhibited migration and invasion. Conversely, ectopic expression of ASF1B accelerated cancer cell migration (*p* < 0.01, Fig. S2e, S2f) and increased invasiveness in cervical cancer cells (*p* < 0.01, Fig. S2g, S2h).

### Knockdown of ASF1B in cervical cancer cells slowed tumor growth in the recipient mice

**Fig. 3:**
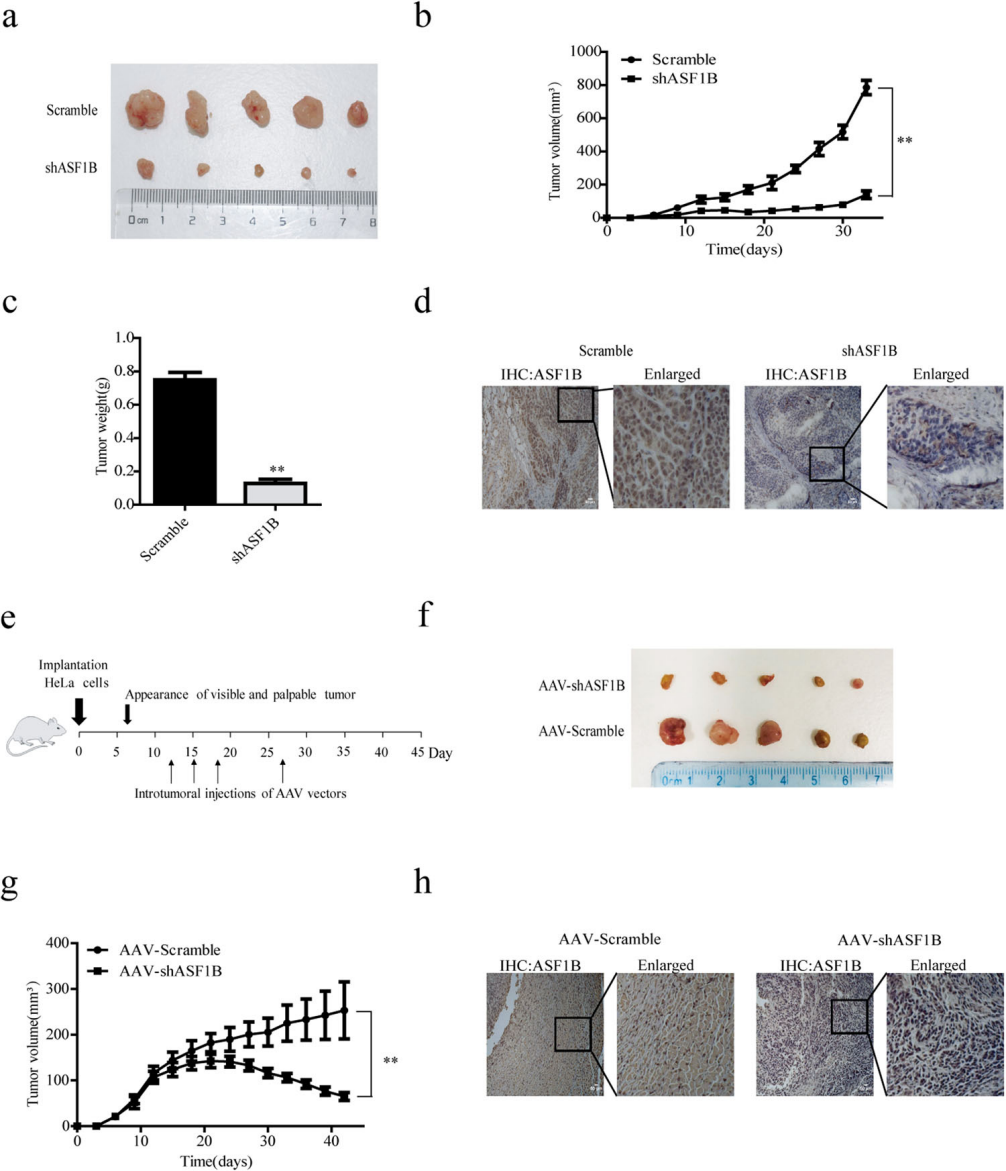
Knockdown of ASF1B in HeLa cells or xenograft tumors in vivo following AAV-shRNA-ASF1B administration suppressed tumor growth. Stable ASF1B-shRNA-HeLa cells, as well as their relative controls, were prepared and harvested. A total of 5 × 10^6^ cells were injected subcutaneously into the flank of each nude mouse. Tumor sizes were measured every three days. **a** Images of tumors after each mouse was euthanized. **b** Measured tumor volumes. The data show that the ASF1B-shRNA tumors grew more slowly than the scrambled control tumors. **c** Accumulated tumor weights are shown for mice receiving ASF1B-shRNA HeLa cells and control cells. **d** The tumors were harvested and then fixed with formalin to prepare slides. IHC was performed to detect the expression of ASF1B. **e** A total of 5 × 10^6^ HeLa cells were injected subcutaneously into the flank of each nude mouse. AAV-shRNA-ASF1B or AAV-scrambled was injected into the formed tumors, and growth was observed on days 12, 15, 18, and 27. **f** Images of tumors after each mouse was euthanized. **g** Measured tumor volumes. The mean volume of tumors after administration was significantly smaller for the AAV‑shRNA-ASF1B group than for the scrambled AAV group. **h** The tumors were harvested and then fixed with formalin to prepare slides. IHC was performed to detect the expression of ASF1B.

ASF1B downregulation inhibited cell growth and migration in cervical cancer cells in vitro, which encouraged us to investigate the effects of ASF1B on tumor growth in vivo. To this end, 5 × 10^6^ ASF1B-shRNA-HeLa or scrambled control cells were injected subcutaneously into the flank of each nude mice. Tumor sizes were measured every three days. The measured data showed that the tumors grew more slowly than those of the scrambled control, and this difference increased until the endpoint of 7 weeks (Fig. 3a, b). Consistently, ASF1B-shRNA-HeLa tumors were smaller in size and lower in weight compared to scrambled control tumors (Fig. 3c). IHC results indicated that ASF1B expression was decreased in ASF1B-shRNA tumors compared to that in control tumors (Fig. 3d).

### Xenograft tumors were reduced in vivo following AAV-shRNA-ASF1B administration

To further explore the effect of ASF1B silencing in vivo, AAV-shRNA-ASF1B was constructed and packed into an AAV virus. Viral titers were determined using qPCR. HeLa cells were harvested and injected subcutaneously into the flank of each nude mouse at a dose of 5 × 10^6^ cells per mouse, and tumor formation was observed. AAV-shRNA-ASF1B or AAV-scrambled at a dose of 5 × 10^11^ viral particles was injected into the formed tumors at days 12, 15, 18, and 27 (Fig. 3e). The mean volume of HeLa-derived tumors after administration was significantly smaller for the AAV‑shRNA-ASF1B treated group (*n* = 5) than for the scrambled AAV group (*n* = 5) (*p* < 0.01, Fig. 3f, g). Interestingly, the average tumor size reached its peak (141.31 mm^3^) 12 days after the administration of AAV‑shRNA-ASF1B, and the tumor size then decreased gradually. Finally, the mean tumor size in the AAV‑shRNA-ASF1B-treated group was only 65.43 mm^3^, while the average size was 253.2 mm^3^ in the control group at their life endpoint. These data further supported the notion that ASF1B silencing led to the inhibition of tumor growth in cervical cancer cells. IHC results also demonstrated the decreased expression of ASF1B in tumors with AAV-shRNA-ASF1B administration (Fig. 3h).

### ASF1B knockdown suppressed HeLa cell proliferation by arresting the cell cycle and regulating the apoptotic pathway

**Fig. 4:**
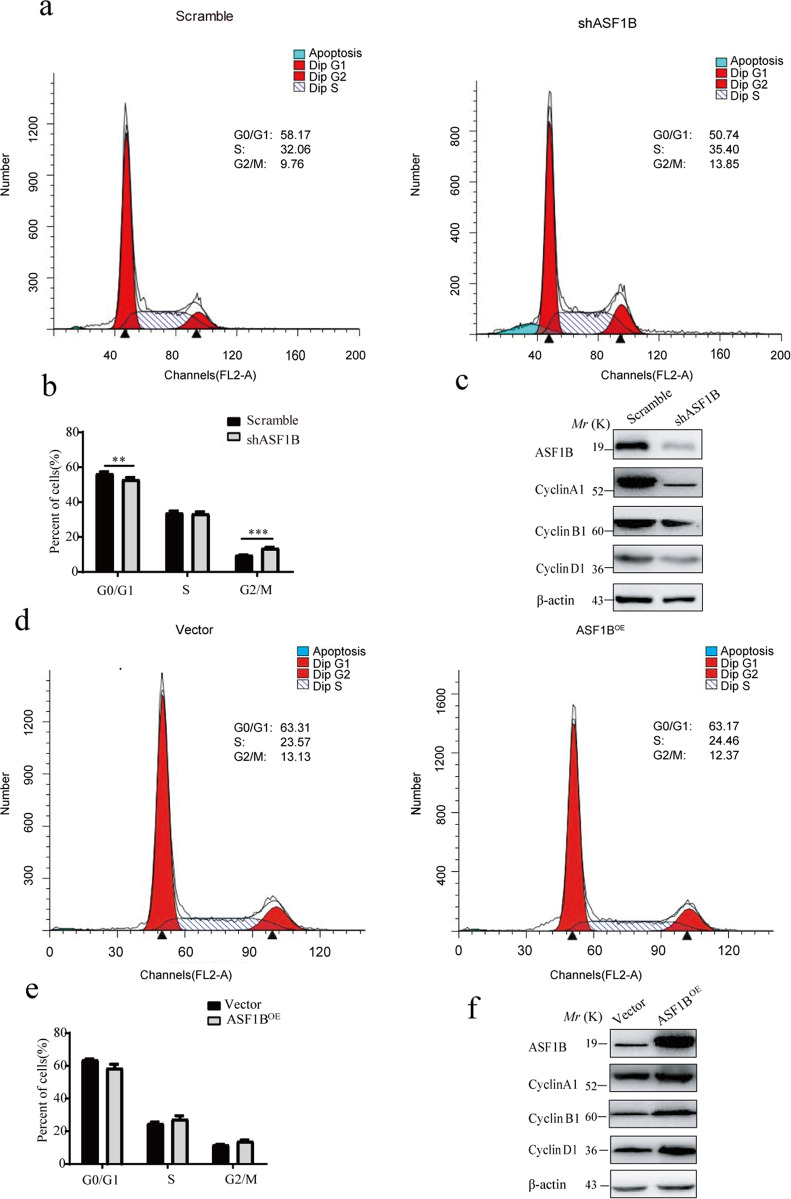
ASF1B knockdown arrested the cell cycle. **a** Flow cytometry was used to detect the cell cycle changes in HeLa cells with ASF1B silencing. More stable ASF1B-shRNA cells remained in the G2/M stage than scrambled cells. **b** Accumulated analysis of the cell cycle. **c** Western blots were performed to detect the cell cycle-related proteins cyclin A1, cyclin B1, and cyclin D1. **d** Flow cytometry was used to detect cell cycle changes in HeLa cells overexpressing ASF1B. No significant cell cycle alterations were found when comparing ASF1B-overexpressing cells and vector cells. **e** Accumulated analysis of the cell cycle. **f** Western blots were performed to detect the cell cycle-related proteins cyclin A1, cyclin B1, and cyclin D1.

To explore the molecular mechanism of the ASF1B knockdown-mediated suppression of cervical cancer cell growth, the cell cycle was also investigated. We found that ASF1B inhibition caused G2 phase cell cycle arrest and significantly inhibited the cell cycle (Fig. 4a, b). Similarly, a panel of well-characterized signaling molecules of the cell cycle was detected in ASF1B-shRNA cells and control cells by western blot. As shown in Fig. 4c, weaker bands for the cyclin A1, cyclin B1 and cyclin D1 proteins were shown in ASF1B-shRNA-HeLa cells compared to those in control cells. These results indicated that knockdown of ASF1B induced cell cycle arrest, which mediated the inhibition of cervical cancer cell growth. We also monitored the cell cycle and the signaling molecules of the cell cycle in ASF1B-expressing HeLa cells. Interestingly, there was no significant change in the cell cycle (Fig. 4d, e), although the bands for the cyclin A1, cyclin B1 and cyclin D1 proteins were increased (Fig. 4f).

**Fig. 5:**
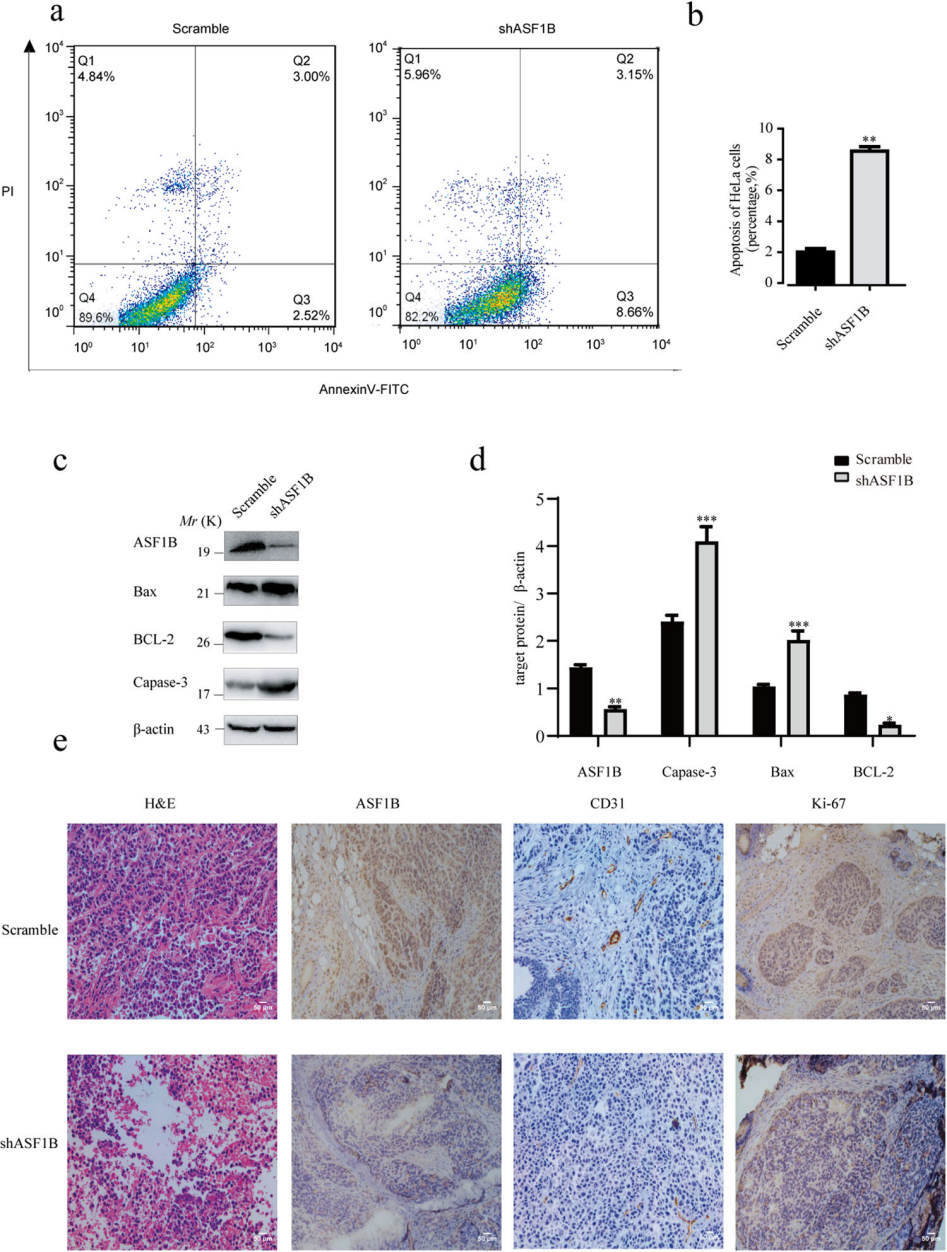
ASF1B knockdown induced apoptotic HeLa cell death by suppressing the Bcl-2/ Bax /caspase apoptotic pathways. **a, b** Flow cytometry was used to detect the apoptosis of HeLa cells with ASF1B silencing. Stable ASF1B-shRNA-HeLa cells and control cells were seeded into 6-well plates. After washing and staining with PE Annexin V and 7-AAD, flow cytometry analysis was performed. **c** Protein expression of canonical molecules important for cell apoptosis. Western blot was used to detect the protein levels of Bax, Bcl-2, and cleaved caspase 3 in the indicated cells. **d** Quantification of the results in (**c**). Data are mean ± SEM, *n* = 3, and two-tailed unpaired Student’s *t* test was used. ***p < 0.001. **e** H&E staining shows the tumor structure; IHC was performed to detect the expression of ASF1B, CD31, and Ki67.

Next, cell apoptosis was also measured to elucidate the molecular mechanism of ASF1B knockdown. The results showed that more cells were apoptotic in ASF1B-shRNA HeLa cells than in scrambled cells (Fig. 5a, b). Next, a panel of apoptotic signaling molecules was examined in ASF1B-shRNA cells and control cells by western blot. As shown in Fig. 5c, d, stronger bands for Bax and cleaved caspase 3 and a weaker band for Bcl-2 were detected in ASF1B-shRNA-HeLa cells compared to those in control cells, suggesting that knockdown of ASF1B induced apoptotic cell death.

Ki67 is a well-known proliferation marker for evaluating cell proliferation^38^, and CD31 is a useful marker for evaluating tumor microvessel density^39^. Thus, we also performed IHC staining for Ki67 and CD31. IHC staining demonstrated decreased Ki67 and CD31 levels in tumors with AAV-shRNA-ASF1B administration compared to those in tumors with AAV-scrambled treatment (Fig. 5e).

### CDK9 was involved in the progression of ASF1B-mediated HeLa cell proliferation

**Fig. 6:**
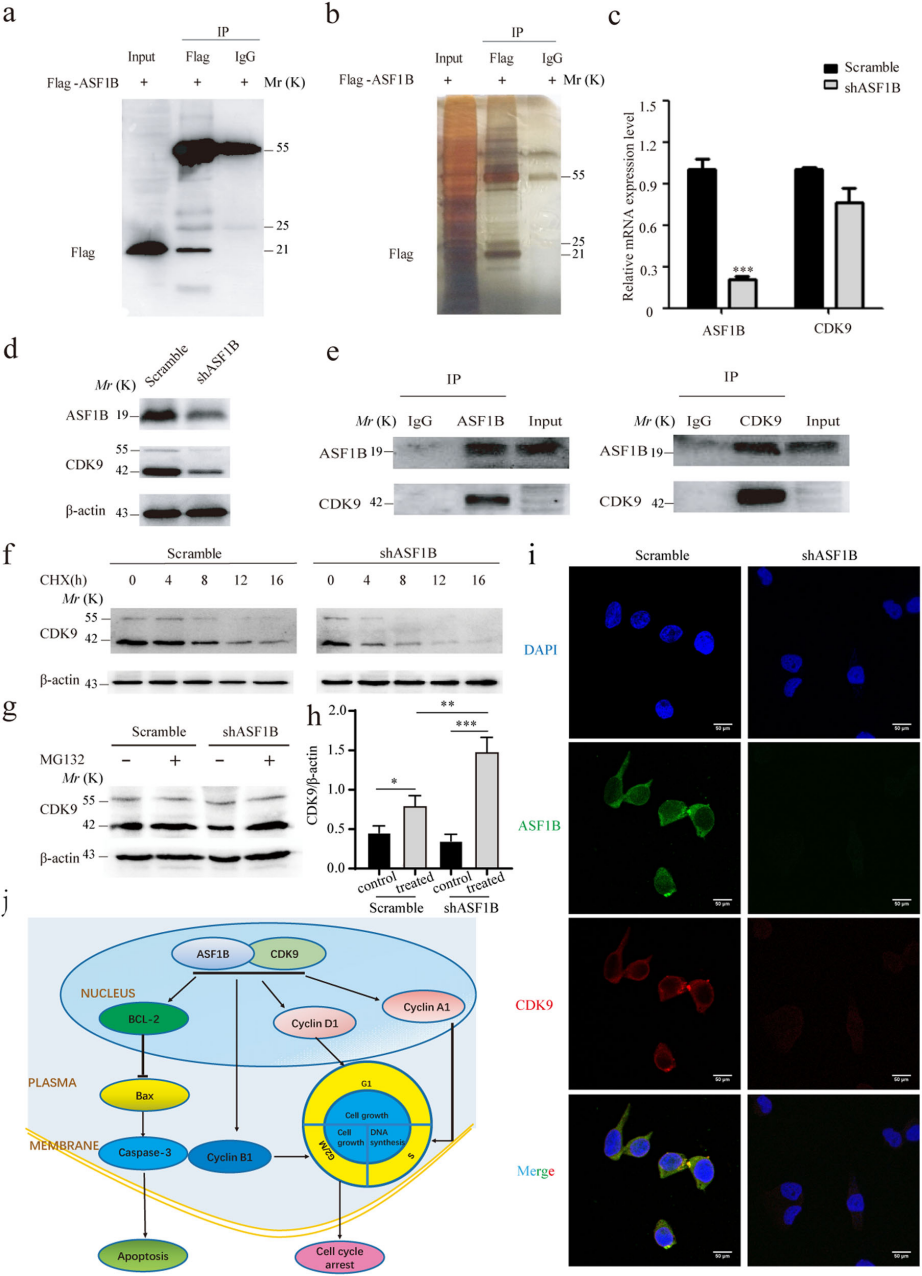
Interaction of ASF1B and CDK9. **a** Western blots were performed to confirm the IP efficiency. **b** SDS-PAGE was conducted to analyze the production of IP. **c** Levels of CDK9 mRNA expression in stable ASF1B-shRNA HeLa cells. **d.** Protein expression of CDK9 in stable ASF1B-shRNA HeLa cells. e. Co-IP was performed to detect CDK9-ASF1B using anti-ASF1B beads and anti-CDK9 beads. **f** ASF1B-shRNA-HeLa cells or scrambled cells were treated with CHX at 20 μg/ml for 0, 4, 8, 12, and 16 h, and then the CDK9 expression level was determined by western blot. **g** ASF1B mediates stabilization through the ubiquitin proteasome pathway. ASF1B-shRNA HeLa cells or control cells were treated with proteasome inhibitor MG132 at 5 μM for 4 h, and then the CDK9 expression level was examined by western blot. **h** Quantification of the results in (**d**). Data are mean ± SEM, *n* = 3, and two-tailed unpaired Student’s *t* test was used. ***p < 0.001. **i** Colocalization of ASF1B and CDK9 in the nucleus. Immunofluorescent staining and imaging were used to visualize the colocalization of ASF1B (green fluorescence) and CDK9 (red fluorescence) in stable ASF1B-shRNA HeLa cells and corresponding scrambled cells. **j** A schematic model of this work. A schematic diagram showing the signaling pathway of the ASF1B-mediated effect on cervical cancer cell growth via the ASF1B/CDK9 axis.

To further explore the mechanism of ASF1B, LC-MS was performed to screen the interaction proteins of ASF1B. Ectopic ASF1B-expressing HeLa cells were extracted for IP, and western blot was conducted to verify IP efficiency (Fig. 6a). IP products were subjected to SDS-PAGE electrophoresis (Fig. 6b) and LC-MS. LC-MS results showed that some proteins could bind to ASF1B, including MD2H, FOXF1, and CDK9 (Table 2). Interestingly, we found that CDK9, which is required for gene silencing^40^ and plays an important role in the pathogenesis^41,42^ in cancer cells, was involved, and we speculated that CDK9 might be an important transcription factor for ASF1B to mediate the occurrence and progression of cervical cancer. We subjected the ASF1B and CDK9 genes to the website (http://genemania.org/) to predict the relationship between these two proteins. The results indicated that ASF1B could bind CDK9 via ASF1B-MSH2-CDK9, ASF1B-KHSRP-CDK9 or ASF1B-HIRA-CDK9 (Fig. S3). Following, we detected the mRNA and protein levels of CDK9 in stable knockdown ASF1B cells and scrambled cells. To our surprise, ASF1B knockdown reduced CDK9 protein expression, but the mRNA level of CDK9 was not altered in stable ASF1B-shRNA cells (Fig. 6c, d). Co-IP was then performed with anti-ASF1B or anti-CDK9 antibodies using normal HeLa cells. Figure 6e shows that endogenous ASF1B formed stable complexes with CDK9.

**Table 2:** The results of proteome analysis.

Gene name	Description	Mol. weight [kDa]	iBAQ exp	iBAQ igg
ASF1B	Histone chaperone ASF1B	22.433	7619100	0
MDH2	Malate dehydrogenase, mitochondrial	35.503	1319400	0
FGFR1OP	FGFR1 oncogene partner	38.099	1285500	0
PPP2R1A	Serine/threonine-protein phosphatase 2A 65 kDa regulatory subunit A alpha isoform	65.308	216090	0
PRRC2A	Isoform 2 of Protein PRRC2A	227.84	164310	0
RPS17	40S ribosomal protein S17	15.55	1160000	0
FOXF1	Fork head box protein F1	40.122	836220	0
DHX29	ATP-dependent RNA helicase DHX29	155.29	46364	0
SSSCA1	Sjoegren syndrome/scleroderma autoantigen 1	21.474	1948100	0
ENO1	Alpha-enolase	47.168	323990	0
PCM1	Pericentriolar material 1 protein	210.13	52791	0
PRDX5	Isoform Cytoplasmic peroxisomal of Peroxiredoxin-5, mitochondrial	17.031	724200	0
RAVER1	Ribonucleoprotein PTB-binding 1	77.843	101720	0
FMR1	Isoform 4 of Synaptic functional regulator FMR1	68.454	256320	0
YBX3	Isoform 2 of Y-box-binding protein 3	31.947	989920	0
HNRNPC	Heterogeneous nuclear ribonucleoproteins C1/C2	25.256	711470	0
ENO3	Beta-enolase (Fragment)	30.402	152620	0
LDHB	L-lactate dehydrogenase (Fragment)	25.218	100620	0
CDK9	Cyclin-dependent kinase 9	42.777	129950	0
CPS1	Isoform 2 of Carbamoyl-phosphate synthase [ammonia], mitochondrial	116.04	15703	0
DHX36	ATP-dependent RNA helicase DHX36 (Fragment)	91.43	20909	0
EEF1G	Elongation factor 1-gamma	50.118	76861	0
GPI	Glucose-6-phosphate isomerase (Fragment)	64.824	473600	0
IQSEC1	IQ motif and SEC7 domain-containing protein 1	91.997	52195	0

To further elucidate the underlying mechanism of ASF1B and CDK9 in cervical cancer progression, we hypothesized that ASF1B knockdown reduces CDK9 protein levels by promoting its degradation. CHX, a de novo protein biosynthesis inhibitor, was used to treat stable ASF1B knockdown cells or scrambled cells. We found that compared with the vector control, ASF1B knockdown reduced the stability of CDK9 protein (Fig. 6f). Treatment with MG132 induced to an increase in CDK9 levels in ASF1B-shRNA HeLa cells compared to control cells (Fig. 6g, h). Together, these data demonstrated that ASF1B promote proteasomal stabilization of CDK9.Then, immunofluorescent staining and imaging were used to visualize the colocalization of ASF1B and CDK9 in stable ASF1B-shRNA HeLa cells and corresponding scrambled cells. The co-staining images of ASF1B (green fluorescence) and CDK9 (red fluorescence) indicated that ASF1B was present in the nucleus and co-localized with CDK9 in scrambled cells, and the immunofluorescent signal of CDK9 was also weak in the nucleus following ASF1B knockdown (Fig. 6i)^43^.

Taken together, these results suggest that impaired expression of ASF1B inhibits cervical cancer growth and induces apoptosis, which is associated with modulation by the ASF1B/CDK9 pathways (Fig. 6j).

## Discussion

Although some biomarkers, such as SSC-Ag, CA-125, CEA, and cytokeratin, have been reported as markers of cervical cancer^44^, the lack of progress in early diagnosis and treatment reveals the urgent need for increased efforts in cervical cancer research. Previous studies evaluating the effect of ASF1B on cancers revealed that ASF1B functions as an oncogene to promote tumor growth in breast cancers, cell renal cell carcinoma, prostate cancers^24,34,35^. These studies indicated that the high level of ASF1B was correlated with increased rates of cancer progression and metastasis occurrence. However, very little was found in the literature describing ASF1B as a pivotal oncogenic gene modulating cervical cancer growth.

In the present study, we first evaluated ASF1B mRNA levels in cervical cancer tumor and para-carcinoma tissues and found that aberrantly high expression of ASF1B occurs in cervical tumors, which was confirmed by qPCR and immunohistochemical analysis (Fig. 1a, b). Subsequently, we induced ASF1B silencing and overexpression to investigate its function in cervical cancer progression. We demonstrated that disruption of ASF1B not only suppressed cervical cancer cell growth in vitro but also slowed cervical cancer cell-derived tumor growth in vivo. These works indicated that ASF1B might promote cervical cancer progression.

Using gain- and loss-of-function strategies, we primarily investigated biological behavior changes, such as proliferation, migration, and cell cycle progression in vitro. We detected that knockdown of ASF1B significantly suppressed cervical cancer cell proliferation and clonogenic survival (Fig. S1a, S1c). We also found that ASF1B knockdown effectively inhibited cervical cancer cell migration by wound healing and transwell assays (Fig. S2a, S2c). In contrast, overexpression of ASF1B promoted proliferation (Fig. S1g) and migration (Fig. S2e, S2g). Importantly, in vivo studies indicate that knockdown of ASF1B in cervical cancer cells obviously slowed tumor growth in the recipient mice (Fig. 3a, b). The study of xenograft tumors in vivo with AAV-shRNA-ASF1B administration further supported that ASF1B silencing leads to the inhibition of tumor growth in cervical cancer cells (Fig. 3f, g). These results suggest that ASF1B is crucial for maintaining cervical cancer cell tumorigenic activity in vitro and promoting tumor growth in vivo.

To elucidate the molecular mechanism of ASF1B knockdown-mediated suppression of cervical cancer cells, protein–protein interaction studies were performed by IP and LC-MS to screen protein groups. As shown in Table 2, we listed approximately 23 proteins involved in the gene regulatory networks of ASF1B. To our surprise, we found that CDK9 could bind to ASF1B. CDK9 belongs to the cyclin-dependent kinase family and is a transcriptional activator recruited to promote RNAPII promoter-proximal pause release^40^. Recently, some studies disclosed that CDK9 plays a pivotal role in breast cancer^45^, acute myeloid leukemia^46^, prostate cancer^47^, ovarian cancer^48^, and osteosarcoma^42^. Yamamoto et al. reported that FIT-039, CDK9 inhibitor, could inhibit replication of HSV-1, HSV-2, human adenovirus, and human cytomegalovirus in cultured cells^49^. Xu et al. investigated that CDK9 levels are highly correlated with the FIGO stage, pathological grade, deep-stromal invasion, tumor size, and lymph nodes metastasis and knockdown of CDK9 inhibits cervical cancer cell proliferation in vitro, as well as tumorigenesis in vivo^36^. CDK9 was involved in cancer progression through the BRD4-dependent recruitment of p-TEFb for the transcription of the MYC gene, which is a proto-oncogene controlling cell growth and cell cycle progression^50^. In addition, some papers have reported that CDK9 is also involved in the apoptosis pathway through apoptotic or anti-apoptotic proteins, such as cleaved caspase 3^51^, Bcl2^52^, and Bax^53^. Interestingly, our data also showed that ASF1B suppression caused G2/S stage cell cycle arrest (Figs. 4a, b), and more cells were apoptotic though effects on Bcl2, Bax, and caspase-3 (Fig. 5c). As mentioned in the literature review, the Bcl2/Bax/caspase3 pathway is an important apoptosis pathway^54^, and our results confirmed that ASF1B disruption mediated by shRNA caused cervical cancer cell apoptosis in this way. Subsequently, we further confirmed that ASF1B and CDK9 are the factors of a nuclear complex to promote cervical cancer progression by co-IP and colocalization of ASF1B and CDK9 in the nucleus (Fig. 6e, i).

Post-translation modifications of proteins, including phosphorylation, acetylation and ubiquitination, play a vital role in controlling numerous biologicals, such as the cell cycle and energy metabolism, among others^55^. Normally, protein ubiquitination acts as a targeting signal to induce protein degradation, in part through the regulation of protein–protein interactions^56^. Kiernan et al. reported that CDK9 is ubiquitinated and degraded by the proteasome whereas cyclin T1 is stable. CDK9 ubiquitination was modulated by cyclin T1 and p45^SKP2^. They proposed a novel mechanism whereby recruitment of SCFSKP2 mediated by cyclin T1 while ubiquitination occurs exclusively on CDK9^57^. To investigate whether stability is involved in the relationship of ASF1B-CDK9, we examined CDK9 protein stabilization with CHX treatment. The results showed that CDK9 was an unstable protein with a half-life of 12 h (Fig. 6f). All these results propose a novel mechanism that ASF1B might stabilize CDK9 protein by inhibiting its proteasome-mediated ubiquitination and degradation.

## Conclusions

Our study focuses for the first time on the oncogenic activity of ASF1B required to develop cervical cancer tumorigenesis. Disrupting ASF1B can prominently suppress cervical tumor growth by regulating cell cycle and apoptosis pathways. These findings may offer a potential target for developing an anti-tumor cervical cancer therapeutic strategy.

## Supplementary information

Supplementary Table S1 & Figure legends

Figure S1

Figure S2

Figure S3
