# Supplementary material for: ASF1B promotes cervical cancer progression through stabilization of CDK9
Source: Cell Death Dis. 2020 Aug 26;11(8):705. doi: 10.1038/s41419-020-02872-5 (PMC7449975; doi:10.1038/s41419-020-02872-5)
Supplement: Supplementary file 1 — Supplementary Table S1 & Figure legends [file 41419_2020_2872_MOESM1_ESM.docx]

**Figure S1. Knockdown or overexpression of ASF1B affected cell proliferation and cell colony formation.** CCK-8 assays were used to measure cell viability in stable ASF1B-shRNA HeLa cells (a), ASF1B-shRNA CaSki cells (b), and ASF1B-overexpressing HeLa cells (e) and ASF1B-overexpressing CaSki cells (f). n=3, the error bars represent the mean ± SEM. Colony formation assays were also conducted. Cells were harvested and seeded in 10-cm plates at 300 cells per plate. Fourteen days later, crystal violet staining was used to define colony formation. Representative images of colony formation and the average cell colony numbers are shown for stable ASF1B-shRNA cells (c) and (d) or ASF1B-overexpression cells (g) and (h).

**Figure S2. ASF1B knockdown suppressed cell motility and ASF1B overexpression-induced cell motility according to wound healing and transwell assays.** For wound healing assays, stable ASF1B-shRNA cells or ASF1B overexpression cells, as well as their controls, were seeded into 6-well plates. Next, 2-mm scratches were generated using a 200-µl pipette tip. The cell-free gap was measured at the indicated times. Representative images of cell-free gaps and average widths of the gaps are shown for shRNA-ASF1B cells (a) and (b) ASF1B overexpression cells (e) and (f). For the transwell assays, 50 µg/cm^2^ reconstituted Matrigel was added to polycarbonate filters. Next, 2$\times$10^3^ cells were seeded into the upper chamber with 200 µl serum-free DMEM. DMEM with 10% FBS was added to the lower chamber, and the cells were cultured at 37°C in a 5% CO_2_ humidified atmosphere. Representative images of invaded cells are shown for shRNA-ASF1B cells (c) and (d) and ASF1B overexpression cells (g) and (h). n=3, the error bars represent the mean ± SEM.

**Figure S3. The relationship predetermination between ASF1B and CDK9.** We subjected ASF1B and CDK9 genes to the website (http://genemania.org/) to predict the relationship between these two proteins.

| **Table S1 Oligonucleotide sequences for this study** | | |
| --- | --- | --- |
| Target | Sense (+) Antisense (-) | Sequence (5’-3’) |
| PCR-Primer |  |  |
| ASF1B | + | CCCAAGCTTGCCACCATGGCCAAGGTGTCGGTGCTGAAC |
|  | - | CGCGGATCCCTATTAGATGCAGTCCATGGAGTTCTCAGGGAG |
| qPCR-Primer |  |  |
| ASF1B | + | ACGACCTGGAGTGGAAGAT |
|  | - | GCTGGAGCTGGGAGAAAT |
| CDK9 | + | CCAGAAGCGGAAGGTGAA |
|  | - | CCAGAAGAAGTCGTGGTTGAG |
| GAPDH | + | CCCCTTCATTGACCTTCAACTA |
|  | - | GAGTCCTTACGATACCAAAG |
| shRNA-ASF1B | |  |
|  | + | CACCGCCTGGAGTGGAAGATCATTTCAAGAGAATGATCTTCCACTCCAGGTTTTTTG |
|  | - | GATCCAAAAAACCTGGAGTGGAAGATCATTCTCTTGAAATGATCTTCCACTCCAGGC |
|  |  |  |
