# Supplementary figures and images for: ASF1B promotes cervical cancer progression through stabilization of CDK9
Source: Cell Death Dis. 2020 Aug 26;11(8):705. doi: 10.1038/s41419-020-02872-5 (PMC7449975; doi:10.1038/s41419-020-02872-5)

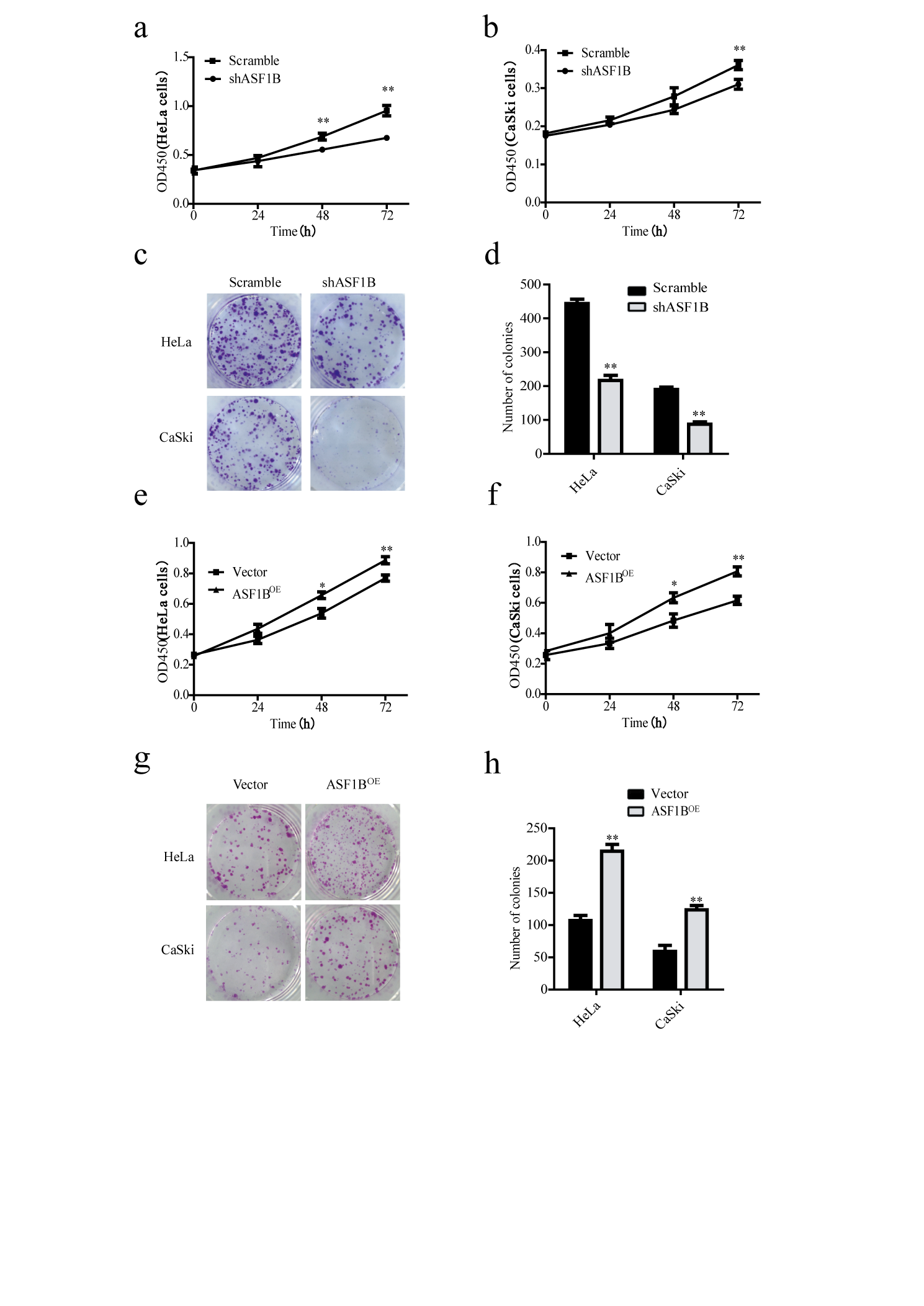

Supplement: Supplementary file 2 — Figure S1 [file 41419_2020_2872_MOESM2_ESM.tif]

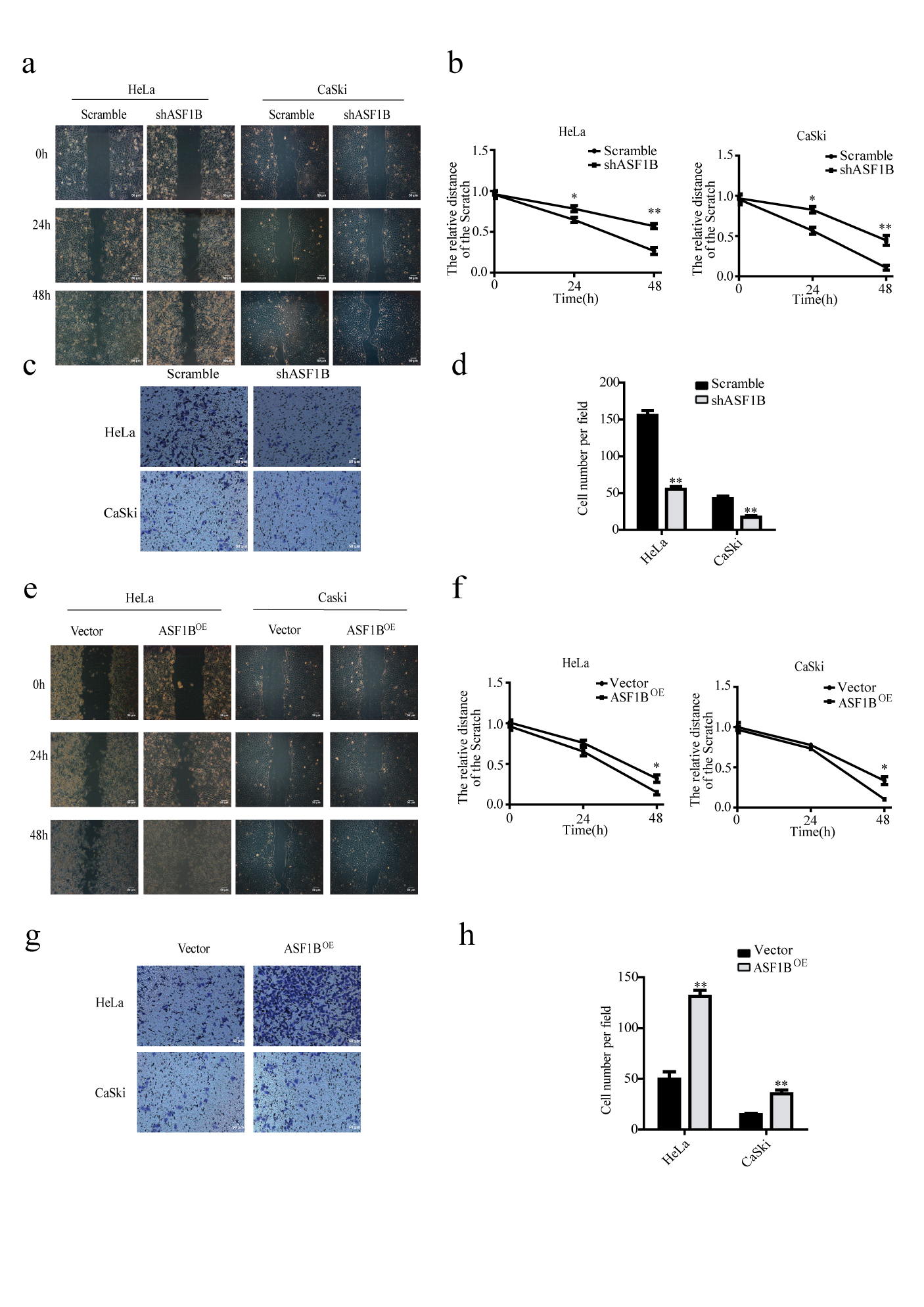

Supplement: Supplementary file 3 — Figure S2 [file 41419_2020_2872_MOESM3_ESM.tif]

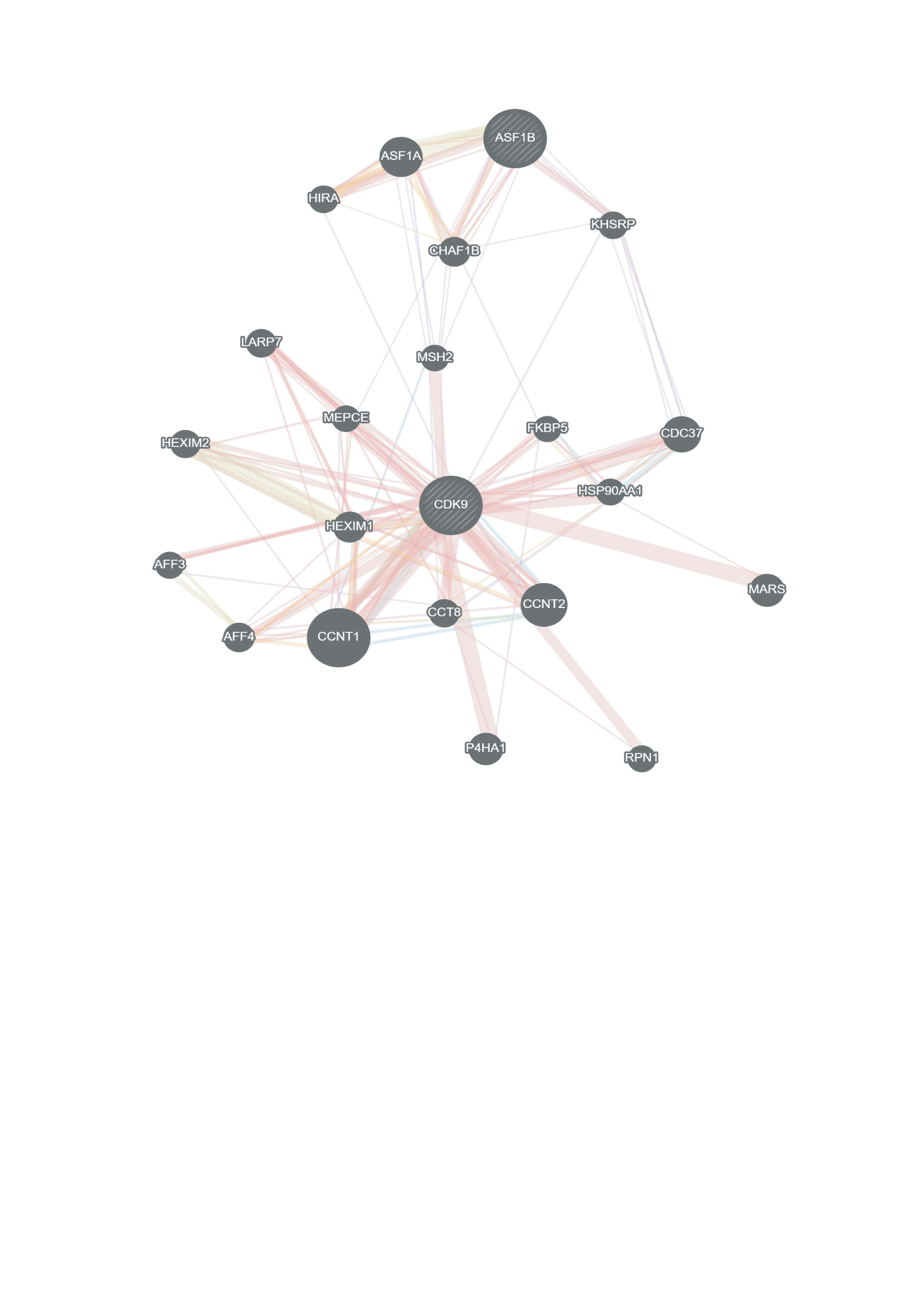

Supplement: Supplementary file 4 — Figure S3 [file 41419_2020_2872_MOESM4_ESM.tif]
